# Potential Role of Photochemistry in Environmental DNA Degradation

**DOI:** 10.1021/acs.estlett.4c00704

**Published:** 2024-11-26

**Authors:** Eliane Ballmer, Kristopher McNeill, Kristy Deiner

**Affiliations:** Institute of Biogeochemistry and Pollutant Dynamics, ETH Zurich, 8092 Zurich, Switzerland

**Keywords:** Photolysis, triplet state excited CDOM, ROS, microbial respiration, biodiversity, eDNA

## Abstract

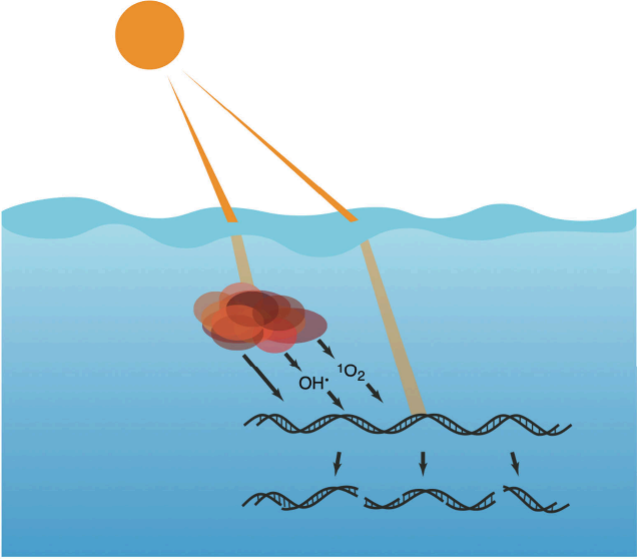

Given the severe loss of species richness across diverse ecosystems, there is an urgent need to assess and monitor biodiversity on a global scale. The analysis of environmental DNA (eDNA), referring to any DNA extracted from environmental samples and subsequently sequenced, is a promising method for performing such biodiversity related studies. However, a comprehensive understanding of the factors that drive distinct eDNA degradation rates under different environmental conditions is currently missing, which limits the spatiotemporal interpretations that are possible from the eDNA-based detection of species. Here, we explore what role photochemistry may play in the fate of eDNA in aquatic ecosystems. Since few eDNA photodegradation studies have been performed, we extrapolate measured photochemical degradation dynamics from dissolved organic matter (DOM) and cellular DNA to what is expected for eDNA. Our findings show that photochemistry may dominate eDNA degradation under certain environmental conditions (e.g., DOM-rich waters with no light-limitation) and that photochemical alteration of eDNA may impact microbial respiration rates and the quantitative polymerase chain reaction (qPCR)-based detection of eDNA. We therefore encourage future studies to analyze the impact of photochemistry on eDNA degradation and provide suggested research directions that could help improve the accuracy of spatiotemporal inferences from eDNA analyses.

## Introduction

The observed loss in biodiversity across various taxonomic groups and geographical locations endangers the health and stability of affected ecosystems.^1,2^ To contain and counteract this loss in species richness, it is essential to understand and globally monitor the changes occurring in biodiversity. Recent studies (see for example refs (3−6)) have demonstrated that the analysis of environmental DNA (eDNA), which refers to any DNA recovered from water, soil or air samples and subsequently extracted and sequenced, is a promising tool for performing such biodiversity related research on a global scale.^3−8^ However, the degradation, transport and sorption processes of eDNA can skew respective measurements and thus the inference of species prevalence.^8,9^ A well-resolved understanding of the fate of eDNA is therefore necessary to make accurate spatiotemporal inferences of species occurrences from eDNA analyses.^8,9^

**Figure 1:**
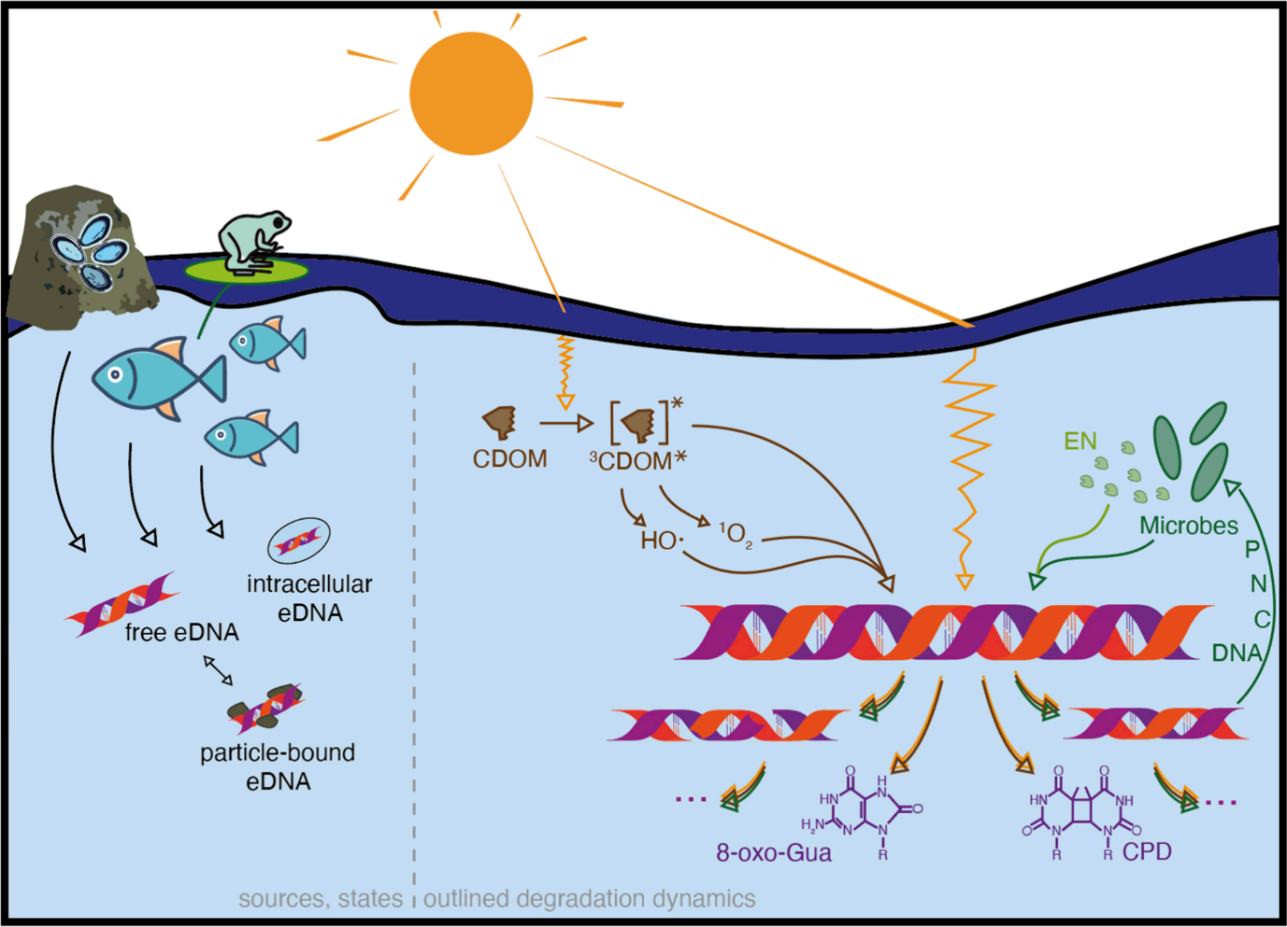
An illustration of potential eDNA sources in aquatic ecosystems, the states eDNA can be found in (i.e., intracellular, free, and particle-bound eDNA) as well as the degradation dynamics of eDNA outlined in this review. From left to right, these degradation processes depict (a) indirect photochemical degradation of eDNA by photochemically excited chromophoric dissolved organic matter (^3^CDOM*) or the thereof produced reactive oxygen species, hydroxyl radicals (HO·) and singlet oxygen (^1^O_2_), (b) direct photodegradation of eDNA via light absorption by eDNA, and (c) microbial utilization of eDNA (i.e., metabolic processes and hydrolysis via extracellular nucleases (EN)). Direct and indirect photodegradation processes are expected to induce single- and double-strand breaks as well as nucleotide modifications (e.g., oxidation of guanine to 8-oxo-Guanine (8-oxo-Gua), dimerization of adjacent thymine nucleotides to a cyclobutane pyrimidine dimer (CPD)), while microbes, with the help of extracellular nucleases, may feed on eDNA for nutrients such as carbon (C), nitrogen (N) or phosphorus (P), or the DNA segments themselves (DNA). Apart from the degradation processes discussed in this review and illustrated in this figure, physical degradation may further contribute to overall eDNA degradation. The AI-assisted drawing tool Beta from Adobe Illustrator was used for illustrating the sources of eDNA (i.e., frog, fish, rock with mussels).

Generally, two sources of eDNA are distinguished: organismal (i.e., DNA contained in intact cells from a whole organism) and extraorganismal eDNA (i.e., DNA no longer associated with multicellular organisms).^10^ This review, however, focuses on extraorganismal eDNA and its dynamics in aquatic ecosystems. Extraorganismal eDNA originates from urine, fecal matter, or epithelial cells shed from alive or the decomposition of dead organisms. After being released into the water column, eDNA may occur in at least the following three states, as illustrated in Figure 1: *Intracellular eDNA* (or *intraorganellar eDNA*) that is contained inside membranes; *free eDNA* that is present in a dissolved state in the water column; and *particle-bound eDNA*, referring to free eDNA adsorbed to particle surfaces.^8−10^ Insight gained from sampling and sequencing all of these different states of extraorganismal eDNA can, as aforementioned, be used in biodiversity related research and has already proven to be useful for biomonitoring, biodiversity assessments and the detection of invasive species.^8,11^

Compared to conventional methods involving the detection and identification of species by direct observations, eDNA-related biodiversity assessment methods are less invasive, easier to perform and allow higher detection probabilities of rare or low-density species.^7^ However, errors such as false positive or false negative detection events limit the spatiotemporal interpretations possible from eDNA analyses.^12^ False positive detections arise if eDNA of a species is detected at a location currently not inhabited by this species due to, e.g., transport processes.^12,13^ False negative detection events describe those cases where eDNA analyses fail to detect the presence of a species at a sampling site it currently inhabits, which may be caused by degradation, transport away or sorption processes removing eDNA from its original location.^8,11,12^

Focusing on eDNA degradation, the overall degradation pathways that may occur in natural environments include physical and photochemical degradation processes as well as microbial utilization of eDNA as nutrient^14^ or DNA source^15,16^ (i.e., metabolic processes or hydrolysis via extracellular nucleases), as has been well summarized by previous literature (see for example Harrison et al.^8^). However, a concise understanding of which factors dominate degradation rates under which environmental conditions has yet to be established. Microbial degradation processes are generally discussed to be the primary driver of eDNA degradation in natural ecosystems, whereby the factors that influence microbial degradation are believed to substantially drive distinct eDNA degradation rates between different ecosystems.^8,17^ However, results from recently published papers put this dominant role of microbial degradation into question, since respective experiments showed that eDNA degradation was influenced by the presence of photochemically excited chromophoric dissolved organic matter (CDOM, i.e., light absorbing and thus photochemically active part of dissolved organic matter (DOM)).^18,19^ This newly appreciated role of CDOM in eDNA degradation highlights the possibility that photochemistry may be more relevant to eDNA degradation than is currently believed.

This is reminiscent of the discussion of the environmental processing and fate of DOM, where scientific consensus initially held that DOM processing was mainly controlled by microbial activity. Over time, this view has shifted as a variety of studies (see for example refs (20−28)) have examined the relative importance of photochemical vs microbial degradation of DOM and found a relevant and sometimes even dominant role of photochemistry in the degradation dynamics of DOM. No comparable body of literature yet exists in the case of eDNA, but there is reason to think that a similar scientific path will be followed given the similarities of DOM and eDNA. Indeed, eDNA itself is a part of the DOM pool and is produced via the same general pathway as other DOM (i.e., by the decomposition of biological material such as algae, plants, or bacteria). We thus anticipate that eDNA may undergo degradation dynamics similar to those of DOM.

Based on the insights obtained from DOM degradation studies, we want to probe the existing narrative of microbial dominance in eDNA degradation and examine whether photodegradation may be more relevant to eDNA degradation than is currently believed. To do so, we review a set of DOM degradation studies that analyzed the photochemical and microbial contributions to overall DOM degradation to understand the role and significance of photochemistry in DOM degradation and set that into the context of eDNA degradation. From there, we shift focus to eDNA photodegradation itself. Since literature and experimental evidence on eDNA photodegradation is limited, we summarize the expected main photodegradation mechanisms of eDNA based on findings from studies that were performed in a biological setting where DNA photodegradation has been extensively studied. We then extend this knowledge to environmental surroundings, including a theoretical evaluation of eDNA photodegradation by photochemically excited CDOM and other photochemically produced reactive species. Subsequently, the obtained conclusions are compared to so-far gathered experimental evidence on eDNA photodegradation to gain insight into how theory relates to practice.

By outlining the expected role of photochemistry in eDNA degradation dynamics and highlighting future study directions, this review emphasizes the essentiality in considering photochemical processes, in addition to biological eDNA degradation, to improve spatiotemporal interpretations of eDNA analyses. Such improvements may eventually enhance the potential of eDNA-based biodiversity assessments to support mitigation activities against biodiversity loss.

## Methods and Materials

### Literature Selection

We classify this review as a State-of-the-art Review according to the taxonomy of Grant and Booth.^29^ The published literature for this review was assembled in stages. First, the relative importance of photodegradation and biodegradation in the overall processing of DOM as well as factors that may determine which degradation pathway dominates were assessed based on existing experimental evidence and set into the context of eDNA degradation. To find the respective studies, search terms such as “DOM photodegradation biodegradation”, “biological photochemical DOM degradation”, or “DOM microbial photochemical degradation” were performed twice, i.e., once in November 2022 and once in September 2023, in Google Scholar. Searches were conducted with both “DOM” and “dissolved organic matter” as search terms. Furthermore, the quantitative assessment of photochemical and microbial contributions to DOM processing presented in Table 1 was performed by reviewing four specific studies that were of comparable experimental design, used similar metrics for quantifying DOM degradation and covered the major climate zones to understand whether different environmental conditions may impact the dominance of either photochemical or microbial degradation processes. The strengths and limitations of using DOM as a proxy for eDNA degradation are discussed in Section “Using DOM as Proxy for eDNA”.

**Table 1:** Summary of the Findings of Amon and Benner,^20^ Obernosterer and Benner,^21^ Cory et al.^22^ and Bowen et al.^23^ on the Relative Importance of Photochemical (PD) vs Microbial (MD) Degradation Processes (i.e., Mineralization to CO_2_) in Overall DOM Degradation Dynamics.^20−23^^a^

DOM source	PD (%)	MD (%)	Key process (−)	r_photo_ (mmol C m^–2^ h^–1^)	r_micro_ (mmol C m^–2^ h^–1^)	DOM type (−)	Climate (−)
Rio Negro (sw)^20^	15	1–3	PD	-	-	(t)	T
Rio Negro (wc)^20^	0.01	0.75	MD	0.41	13.0	(t)	T
Rocky Bluff Swamp^21^	46	27	PD	-	-	t	ST
Lake Murray^21^	7	27	MD	-	-	**t**,a	ST
Phytoplankton^21^	0	74	MD	-	-	a	ST
Imnavait Creek^22^	+	-	PD	1.03	0.1	t	A
White Clay Creek^23^	4.7	24.9	MD	0.06	1.14	**t**,a	TE

a Columns 1 and 2 show the percentage of initially available DOM mineralized by each process (sw = surface water, wc = extrapolated to whole water column), column 3 the relatively more important process (MD, PD), columns 4 and 5 the respective rate constants and columns 6 and 7 the DOM type (a = autochthonous, t = terrestrial, (t) = the respective study did not specify the type of DOM, wherefore it was inferred from the information provided by the study) and the climate zone (T = tropics, ST = subtropics, A = Arctic, TE = temperate).^20−23^ The values in the table are as reported in the source publication. The experimental conditions and analytical methods used by the studies to determine PD and MD are specified in Tables S1 and S2 in the SI.

In the second stage, potential direct and indirect photodegradation mechanisms for eDNA in aquatic ecosystems were identified. To do so, insights from studies on the photodegradation of DNA in a biological context as well as common photodegradation pathways in aquatic ecosystems were combined to outline the expected role and relevance of photochemistry in eDNA degradation. Thereafter, the validity of the elaborated expected role and relevance of photochemistry in eDNA degradation was evaluated based on the so-far gathered experimental evidence on direct and DOM-mediated eDNA photodegradation. Since the respective body of literature was thin, studies performed in the context of antibiotic resistance genes (ARG) were additionally considered. To compile this literature, search terms such as “photodegradation eDNA”, “photodegradation environmental DNA”, “ARG photodegradation”, or “ARG photodegradation DOM” were used in Google Scholar throughout November 2022 and September 2023. The strengths and limitations of using ARGs as proxies for eDNA degradation are discussed in Section “Current Experimental Evidence on eDNA Photodegradation”.

Finally, to gain an overview of studies that examined the phenomenological role of sunlight and/or ultraviolet (UV) light in the degradation of eDNA, searches were conducted in Google Scholar using the search terms ‘(“environmental DNA” or “eDNA”) and “ultraviolet”’ and ‘(“environmental DNA” or “eDNA”) and “sunlight”’ in July 2024. Studies that performed experiments using UVC light were not considered in this search.

### Collection of Ultraviolet–Visible (UV–vis) Absorption Spectra

To assess the relevance of direct photodegradation of eDNA, the UV–vis absorption spectrum of sheared salmon sperm DNA (Invitrogen, 100 mg DNA/L, Longmire buffer) was collected on a Varian Cary 100 Bio Spectrophotometer. Similarly, UV–vis absorption spectra of the DOM isolates Suwannee River Fulvic Acid (SRFA, 3.1 mg C/L, Standard II, 2S101F), Upper Mississippi River Natural Organic Matter (MRNOM, 4.3 mg C/L, 1R110N), Suwannee River Humic Acid (SRHA, 3.7 mg C/L, 2S101H), Suwannee River Natural Organic Matter (SRNOM, 3.9 mg C/L, 2R101N) and Pony Lake Fulvic Acid (PLFA, 4.2 mg C/L, 1R109F) were collected in phosphate buffer (pH = 7) to compare their absorption behavior to that of eDNA.

## From DOM to eDNA

### Using DOM as Proxy for eDNA

As mentioned in the Introduction, the objective of this review is to probe the idea that microbial degradation dominates the fate of eDNA. Since the relative importance of photochemical vs microbial degradation processes has extensively been studied for DOM but not for eDNA, we extrapolate insights gained from DOM to eDNA.

The relevance of using DOM to anticipate the environmental fate of eDNA is given not only by the fact that eDNA itself belongs to the pool of DOM but also by their chemical similarity. Like DOM, eDNA is a nutrient source for carbon, nitrogen and phosphorus for heterotrophic bacteria.^14,15,30^ In addition, DOM is well described as a negatively charged polyelectrolyte and, while likely not composed of macromolecules, DOM has characteristics in common with macromolecules due to its propensity to form supramolecular assemblies.^31^

When using DOM as a proxy for eDNA it is important to consider that the chemical composition and structure of eDNA is well-defined, while DOM is a complex mixture of organic compounds, the exact chemical characterization of which is largely unknown.^32,33^ Furthermore, the composition of DOM may vary depending on the source of DOM,^34^ and with that also the respective degradation dynamics. Accordingly, degradation dynamics of DOM may be more variable within and across different ecosystems compared to eDNA.

Finally, the analytical tools used to quantify eDNA or DOM degradation differ (see Tables S2 and S4 for analytical tools used to assess DOM or eDNA degradation in the studies reviewed here). While the degradation of eDNA is often quantified using quantitative polymerase chain reaction (qPCR), DOM degradation is mostly assessed as mineralization to CO_2_ by monitoring either the dissolved organic or inorganic carbon content. In case of eDNA, not only mineralization but also alterations of eDNA (e.g., oxidation of nucleotides) may lead to negative detection events by qPCR as the amplification efficiency^35^ or the binding of the primers used for qPCR could be affected by these damaged regions. Subsequently, the targeted quantification via qPCR may not be capable of separating eDNA mineralization from alteration and may thus be more sensitive to detecting eDNA degradation compared to the nontargeted approach used for quantifying DOM degradation.

Despite these limitations, the so-far gathered experimental evidence on the relative importance of photochemical vs microbial degradation processes for DOM serves as a valuable starting point to explore the relevance of photochemistry in eDNA degradation dynamics. The extrapolation from DOM to eDNA may further reveal, for which aquatic ecosystems photochemistry of eDNA may be especially relevant to explore, how respective experiments could be designed and which environmental factors should be considered when gathering similar experimental evidence for eDNA.

### Relative Importance of Photochemical vs Microbial Processes in DOM Degradation

To understand the relative importance of photochemical and microbial processing of DOM, we examined studies of DOM degradation in freshwater ecosystems of different climate zones (i.e., tropics, subtropics, arctic, and temperate) and DOM types (i.e., autochthonous and terrestrial) including a laboratory tank study on mineralization of phytoplankton DOM.^20−23^ In all experiments, water samples were taken from the respective ecosystems and exposed to either irradiation or the endemic microbial community in the laboratory. Thereafter, total mineralization of DOM to CO_2_ was measured to quantify photochemical and microbial contributions to the overall observed DOM degradation (see Table S2 for the analytical methods used to quantify DOM mineralization in the respective studies). The locations of the different study sites are indicated in Table 1 and Figure S1 in the Supporting Information (SI) alongside more detailed information on respective experimental conditions in Table S1 (i.e., DOM concentration, light source, irradiation, and incubation temperature and time).

The comparison in Table 1 shows that in three out of seven analyzed scenarios, or two out of five if only natural whole waters are considered, the contributions of photodegradation to total DOM mineralization were higher than those of microbial respiration. The results for Imnavais Creek further underscore this finding, as the photomineralization rate was about 10 times higher than that of microbial respiration (i.e., 1.03 vs 0.1 mmol C m^–2^ h^–1^). Consequently, photodegradation appears to be relevant for DOM processing and may even outcompete microbial DOM respiration under certain environmental conditions.

The observed degradation dynamics for Rio Negro and Imnavait Creek suggest the significance of environmental factors in determining the relative importance of photochemical vs microbial degradation of DOM. The Rio Negro is situated close to the equator, therefore experiencing high sunlight exposure.^20^ Together with the high DOM^20^ and thus potentially CDOM concentration to perform photochemical reactions (see discussion below on impact of DOM/CDOM concentration on relative importance of photodegradation), photodegradation may dominate DOM degradation in Rio Negro surface waters. However, throughout the whole water column, photodegradation may no longer compete with microbial degradation, among other things, probably due to light attenuation by CDOM with increasing water depth. In the case of Imnavait Creek, an Arctic shallow-headwater stream, factors such as rather low rates of microbial respiration, high CDOM concentrations, and maximized DOM exposure to sunlight due to the hydrological characteristics of the water body (i.e., shallow, unshaded, temporally thermally stratified) may explain the dominant role of photodegradation.^22,25^ These two examples highlight the importance of understanding the environmental factors that may determine the dominance of either photochemical or microbial degradation processes for DOM, and with that potentially also for eDNA, which is the focus of the next section.

#### Which Environmental Factors Lead to a Dominant Role of Photochemistry?

According to the literature, the following factors, among others, may impact DOM photodegradation and its relative importance in overall DOM degradation: DOM concentration;^20^ DOM quality^20^ (i.e., origin, chemical composition, CDOM fraction of DOM, light absorptivity of CDOM and photolability of DOM^22,24^); water residence time determined by thermal stratification and discharge volume;^22^ biolability of DOM;^23^ light conditions;^20,22^ and seasonality.^36^

Higher DOM concentrations tend to increase photodegradation rates,^20^ due to a higher availability of CDOM to perform photochemical reactions. This effect may, however, only be observed in substrate-limited systems, i.e., systems where the amount of CDOM determines photodegradation rates and sufficient light is available.^22,24^ As CDOM concentrations increase, the average rate of light absorption by CDOM increases as well, decreasing the depth of light penetration in the water column.^22^ If the rate of light absorption by CDOM increases to an extent, where increasing CDOM concentrations no longer increase DOM photodegradation rates, the system becomes light-limited.^22^ At this point, photodegradation rates of DOM no longer depend on the availability of CDOM, but on the amount of incident light reaching the water surface.^22^ If this concept is applied to eDNA, enhanced photodegradation rates of eDNA may be expected in systems with high CDOM concentrations and no light-limitation.

Thermal stratification and water residence time may further impact photochemical degradation rates of DOM.^22^ Long water residence times increase light exposure of surface water DOM enabling more light absorption and, with that, enhanced photochemical degradation rates, especially in light-limited systems.^22^ Consequently, near-surface DOM experiences a higher photochemical impact compared to DOM further down in the water column in thermally stratified water bodies. Since Imnavait Creek is characterized by such periods of thermal stratification, Cory et al.^22^ analyzed the chemical composition of DOM in surface and bottom waters during these stratification periods and found their composition to be significantly different.^22^ Upon light exposure of “bottom water DOM” in the lab, similar characteristics as those of “near-surface DOM” were achieved, confirming that the observed differences in chemical composition were related to photochemical processes.^22^ Stratification periods as well as the transport of DOM to greater water depths may therefore protect DOM from further photodegradation,^24^ especially if most of the UV light reaching the water surface is attenuated by the surface water layer.

If this can be extended to eDNA, eDNA further down in the water column will be protected from photodegradation compared to eDNA found in shallower water layers. Moreover, the influence of thermal stratification on the extent of photodegradation experienced may impact species-specific detection via eDNA analyses. Littlefair et al.^37^ showed that during thermal stratification periods, species-specific eDNA was primarily present in the water layer preferred by the respective species, while it was homogeneously distributed over the whole water column during periods of lake turnover.^37^ In stratified water bodies, eDNA detection of species preferring shallower over deeper water layers may thus be lower if photodegradation rates are high enough to significantly impact total eDNA degradation rates. Considering these findings together with the fact that a significant share of eDNA sampling is conducted in the photic zone (i.e., the upper water layer receiving sunlight), it is worth exploring whether a variation in sampling depths would impact detection probabilities of species from eDNA.

Finally, experiments by Bowen et al.^23^ indicate that photodegradation is not able to compete with microbial respiration when DOM fractions are very labile and therefore easily consumed by microbes.^23^ Environmental DNA is more comparable to the labile DOM fraction, which would suggest a similarly small importance of photochemical processes for total eDNA degradation. However, under conditions of limited microbial activity, such as low enough temperatures to slow microbial processes or high UV irradiation, photodegradation may become a dominant degradation pathway for eDNA.

A study performed by Song et al.^36^ exemplifies this last statement and simultaneously highlights the importance of light conditions and seasonality in determining which degradation process dominates overall DOM degradation.^36^ In their study, they analyzed the seasonal effect on the relative importance of microbial and photodegradation of DOM in the Heilongjiang River in Northeast Asia.^36^ Their results showed that bacterial degradation dominated DOM processing in summer, while photochemical degradation was the primary degradation pathway in autumn and winter, which was linked to differences in temperature, type of DOM input and light intensity.^36^ Such seasonal variations may also apply to eDNA degradation dynamics and thus deserve greater attention in future studies.

### How Does Photochemistry Impact Microbial Degradation?

So far, we have considered photodegradation as a competitor to microbial respiration for only DOM or eDNA as substrates. In the case of DOM, photodegradation not only competes with microbes for DOM as substrate, but can also lead to its chemical alteration, which can either positively or negatively impact the availability of DOM for microbial respiration.^21^ If biodegradable DOM is consumed or becomes more refractory upon photochemical transformation, a decrease in microbial respiration rates of DOM is expected.^21^ On the other hand, microbes could benefit from DOM photochemistry if DOM is transformed into more bioavailable fractions after being exposed to irradiation.^21^ Positive effects have been observed for the more refractory terrestrial DOM, which generally consists of oxygen-rich aromatic and high molecular weight compounds, while negative effects seem to dominate for less refractory, autochthonous DOM abundant in nitrogen rich amino acid-like substrates.^21,24^ It is therefore suggested that the effect of irradiation on DOM’s bioreactivity is inversely coupled to its initial bioreactivity (see Cory and Kling^24^ for mechanistic reasoning).^21,24^

Also, in the case of eDNA, photochemical processes may not only induce photomineralization but also cause photoalterations such as oxidative damage to eDNA nucleotides. Photochemically altered eDNA may show decreased microbial respiration rates, as constituents of eDNA fueling microbial respiration may be photochemically removed or damaged, leaving eDNA less valuable for microbes. Moreover, photoaltered eDNA may no longer be recognized by microbial enzymes responsible for the breakdown of eDNA. Both responses would lead to a higher preservation of photochemically altered eDNA compared with its original state, which would be especially relevant in environments characterized by high photochemical impact. This eventuality contradicts the hypothesis from before, where exactly these environments were suspected to show decreased eDNA persistence due to higher photochemical degradation rates. It is therefore an open question whether photochemistry indeed impacts microbial respiration of eDNA and, if so, whether it primarily leads to the enhanced breakdown (i.e., fragmentation, mineralization) or slowing of microbial utilization due to chemical alterations of eDNA.

Apart from microbial utilization, results by Sikorsky et al.^35^ suggest that photoalterations may impact the detection of eDNA by qPCR.^35^ In their study, they found that DNA base modifications distant from the primer binding sites reduced the qPCR amplification efficiency of a 90-base oligonucleotide.^35^ These base modifications included, for example, the cyclobutane thymidine dimer (TT dimer, effect observed with one base modification) or the oxidation product of deoxyguanosine (8-oxo-dG, effect observed upon juxtaposition of two base modifications), which can also form upon DNA exposure to UV light.^35,38^ If the amplification efficiency and potentially also the binding of PCR primers would significantly be affected by photoalterations of eDNA, the detection efficiency and thus the accuracy of eDNA analyses could be reduced in environments experiencing high photochemical impact. This eventuality reemphasizes the need to assess the dominant photoproducts of eDNA as well as the effect of photoalterations on eDNA detection by qPCR.

## Shifting Focus to eDNA Photochemistry

The discussion above highlights the potential importance of photochemistry in overall DOM processing and particularly in that of eDNA. Since eDNA belongs to the pool of DOM, these insights suggest a similar role and relevance of photodegradation in eDNA degradation dynamics. The rest of this review is thus devoted to the evaluation of the role of photochemistry in eDNA degradation dynamics.

As mentioned in the Introduction, this evaluation includes a summary of the expected photodegradation pathways based on the findings from studies performed in a biological setting and extending those to environmental surroundings, including a theoretical evaluation of eDNA photodegradation by photochemically excited CDOM and thereof produced reactive species. To understand how our theoretical evaluation relates to practice, the obtained conclusions are finally compared to the so-far gathered experimental evidence on the photodegradation of eDNA. This second part of the review thus shifts focus not only to eDNA photochemistry but also from using DOM as a proxy for eDNA to evaluating DOM, or more precisely CDOM, as a reaction partner with eDNA.

### From Biological to Environmental Systems

From the photochemistry studies performed in a biological context, it is well-known that sunlight induces DNA damage via direct and indirect photochemical reactions.^39,40^ Direct photodamage takes place when chromophoric centers within the DNA absorb light, while indirect photodamage occurs upon light absorption by photosensitizers present in the proximate environment of the DNA.^39,40^ These excited photosensitizers may then either directly interact with DNA to degrade it or generate other reactive chemical species that can damage DNA.^39,40^ Among these reactive species are reactive oxygen species (ROS) such as hydroxyl radicals (HO·) and singlet oxygen (^1^O_2_), which we will focus on in this review.^40−42^

Direct photodegradation of DNA most commonly induces dimerization reactions of adjacent pyrimidine nucleotides (i.e., cytosine, thymine), which primarily lead to the formation of the cyclobutane pyrimidine dimers (CPD) but also pyrimidine 6–4 pyrimidone photoproducts and respective Dewar isomers.^38−40,42^ Furthermore, direct photodamage may induce single- (i.e., nicks) or double-strand breaks (i.e., fragmentation) of the DNA backbone.^40^

Indirect photodegradation mediated by excited state photosensitizers or other reactive species mainly occur via energy or electron transfer reactions.^39,43^ Energy transfer reactions from an excited state photosensitizer to the DNA primarily induce dimerization reactions of adjacent pyrimidine nucleotides eventually forming the CPD photoproduct.^43^ Oxidation reactions by either the excited state photosensitizer itself or ROS lead to the oxidation of DNA nucleotides and the deoxyribose backbone and to a lesser extent to single- or double-strand breaks.^39,43^ The purine base guanine is particularly susceptible to these oxidation reactions, since it has a lower reduction potential compared to the other nucleobases.^43−45^ It is most commonly transformed to the 8-oxoguanine photoproduct, the most frequent oxidative DNA lesion.^39,40,43^

Since DNA mostly absorbs in the UVB region (280–320 nm), UVB is considered to be the most relevant wavelength range for inducing direct photodamage (see Figure S2 for eDNA absorption spectrum).^39,46^ Despite the fact that UVA (320–400 nm) absorbance by DNA is much lower, UVA may still induce direct photodamage, since UVA irradiance at the Earth’s surface is 20-fold higher compared to that of UVB.^40^ Concerning indirect photolysis, the relevant wavelength range depends on the absorption spectrum of the photosensitizer.

These photochemically induced DNA degradation pathways are not only relevant in terms of biological systems but can also impact the degradation of eDNA. As natural sunlight meets aquatic ecosystems, free, intracellular/-organellar, and particle-bound eDNA can absorb UV light leading to its direct photochemical degradation. The extent of direct photodegradation will depend on the overlap of the eDNA absorption spectrum and the light spectrum present in the water column, which is shaped by factors such as the incoming solar radiation (i.e., solar zenith angle, time of day, cloud coverage, etc.) and the depth of light penetration into the water column.^47,48^ The latter is determined by the presence of compounds that compete for photons with eDNA by absorbing, scattering and thus attenuating part of the incoming light (e.g., water, DOM, particulate organic matter (POM)).^47,48^ Finally, direct photodegradation rates of eDNA could depend on the state eDNA can be found in (i.e., free, intracellular/-organellar, or particle-bound eDNA), as has been observed for free and particle-bound eDNA by Scappini et al.^49^

Apart from direct photodegradation, photosensitizers present in aquatic ecosystems may trigger indirect photochemical degradation pathways of free or particle-bound eDNA. As discussed for direct photodegradation, the rates of indirect photodegradation reactions may differ between free and particle-bound eDNA. The particle the eDNA is bound to could, for example, slow down indirect photodegradation reactions by physically shielding eDNA from photosensitizers or, if the particle can act as photosensitizer itself (e.g., POM, CDOM), enhance degradation reactions due to the close proximity of the eDNA to the photosensitizer.

In aquatic environments, CDOM is one of the most important photosensitizers.^50^ Its optical characteristics vary depending on the concentration and composition of the CDOM but are generally characterized by an exponential decrease in light absorption with increasing wavelength and, like DNA, CDOM absorbs strongly in the UVB range (see Figure S3 for exemplary absorption spectra of CDOM).^46,51^ Upon photon absorption, a fraction of the CDOM reaches a photochemically excited triplet state (^3^CDOM*), which is considered to be more relevant than excited singlet states due to the longer lifetime of ^3^CDOM*.^50^

In degradation reactions, ^3^CDOM* can directly react with compounds to degrade them, or via the formation of reactive species such as ROS.^50^ When directly interacting with compounds, ^3^CDOM* mostly reacts as an oxidant via electron transfer.^50^ Given what is currently known about the redox behavior of ^3^CDOM* and the production of ROS in natural waters, we have performed a theoretical evaluation of reactions of free eDNA with ^3^CDOM*, HO·, and ^1^O_2_ in the following section.

### Evaluation of ^3^CDOM* and ROS Participation in eDNA Degradation

#### Direct Interaction of ^3^CDOM* with eDNA

The potential reactivity of ^3^CDOM* toward oxidation of eDNA nucleotides can be evaluated by comparing the one-electron reduction potential of naturally occurring ^3^CDOM* to those of DNA nucleotides. The one-electron reduction potential of ^3^CDOM* is estimated to lie in the range of 1.36 to 1.95 V or even 1.6 to 1.8 V (vs NHE).^50,52^ For guanine, adenine, thymine, and cytosine, Faraggi et al.^44^ found one-electron reduction potentials of 1.04, 1.32, 1.29, and 1.44 (vs NHE, pH 7).^44^ These values should be taken as approximate, as a variety of reduction potentials exist for nucleotides in the scientific literature.^45^ As reduction potentials of DNA nucleotides are either below or at the lower end of the range of those of ^3^CDOM*, the oxidation of eDNA nucleotides by ^3^CDOM* should occur from a thermodynamic point of view. Of course, these reactions must be fast enough, and contributions would have to be in a similar order of magnitude as observed for other degradation processes to substantially impact eDNA degradation. Nonetheless, based on this thermodynamic evaluation, we expect ^3^CDOM* to participate in eDNA degradation.

We further expect the eDNA degradation rates to vary as a function of the DOM source. A study by Zhou et al.^53^ for example showed that the charge of the substrate influences the respective degradation rate by ^3^CDOM*, since negative charge may decrease reaction rates due to repulsive effects between anionic substrates and the mostly negatively charged CDOM constituents.^53^ When evaluating various DOM isolates, Zhou et al.^53^ found that for the autochthonous DOM source Pony Lake fulvic acid (PLFA) and for wastewater influenced surface waters (WWOM) reaction rates were similar for neutral and negatively charged substrates, while rates were significantly lower for negatively charged substrates in the case of terrestrial DOM.^53^ PLFA and WWOM contain fewer negatively charged species compared to terrestrial DOM, which could explain this finding.^53^ Based on these observations and the fact that DNA is mostly negatively charged, we expect eDNA photooxidation by ^3^CDOM* to be enhanced in waters characterized by a high input of autochthonous DOM or wastewater.

#### ROS-Mediated Interaction of ^3^CDOM* with eDNA

Apart from direct interactions with substrates, ^3^CDOM* can, as mentioned before, also indirectly participate in transformation reactions of substrates via the formation of reactive species such as ROS, carbonate radical anions (CO_3_^.**-**^) or other transient species (e.g., Br_2_^.**-**^, ·NO_2_).^50,53,54^ In the following section, we focus on the participation of the two ROS, HO· and ^1^O_2_, in eDNA degradation since these two reactive species have proven to be important in aquatic photochemistry and photobiology studies have shown their participation in DNA degradation reactions.^39,43,55^

^**1**^**O**_**2**_. While the general reactivity of ^1^O_2_ is rather low, ^1^O_2_ can play a vital role in degradation processes of selected compounds such as DNA.^54,56^^1^O_2_ is formed by the energy transfer from ^3^CDOM* to molecular oxygen.^54^ The concentrations of ^1^O_2_ in the bulk aqueous phase are low (6 × 10^–17^ to 5 × 10^–15^ M^57^) due to the efficient deactivation of ^1^O_2_ by collision with water molecules.^54^ However, elevated ^1^O_2_ concentrations of 30 to 1500 times are experienced by molecules that are bound to CDOM molecules.^56,58^ This higher near-CDOM ^1^O_2_ concentration may enhance ^1^O_2_-induced transformations of CDOM-bound substrates, which has been shown to be the case for the photochemical ^1^O_2_ inactivation of viruses sorbed to organic matter.^46,56,58^ As mentioned in the introduction, eDNA exists in different states in the environment including particle- or organic-matter-bound eDNA. Since ^1^O_2_ has been shown to effectively oxidize guanine in biological studies, we expect the same to happen to eDNA in aquatic environments especially when bound to DOM or POM. With steady-state concentrations assumed to be in the range of 10^–17^ to 10^–15^ M and bimolecular rate constants on the order of 10^3^ to 10^7^ M^–1^ s^–1^ (see for example refs (19, 59−61)), rate constants of 10^–14^ to 10^–8^ s^–1^ (t_0.5_ ∼ years to millennia) may be expected.

**HO**·. HO· is among the most reactive species involved in aquatic photochemistry and respective reaction kinetics are therefore often close to diffusion-limited.^54,62^ The precise formation mechanism of HO· by photoreaction of CDOM has not yet been fully resolved in contrast to the pathway involving photolysis of nitrate and nitrite.^54,63^ Since CDOM is much more efficient at absorbing light compared to nitrate and nitrite, CDOM may, however, be a significant source for HO· radicals in aquatic ecosystems.^54^ Due to its high reactivity, HO· is effectively scavenged by compounds in the water column resulting in low ambient steady-state concentrations (10^–18^ to 10^–16^ M).^54^ Similar to what has been discussed for ^1^O_2_, Yan et al.^64^ have suggested that HO· steady-state concentrations may be elevated inside the microenvironment of CDOM.^64^ Depending on the substrate, its location as well as the environmental conditions, HO· radicals may thus be more or less important for degradation reactions.^54^ For DNA as substrate, physiological studies have shown HO· to be highly reactive toward DNA by inducing strand breaks as well as oxidative modification of nucleotides.^62,65^ We therefore expect HO· produced by photoreaction of CDOM in aquatic ecosystems to induce similar damage to eDNA, especially when HO· is generated in close proximity to eDNA. Assuming steady-state concentrations in the range of 10^–18^ to 10^–16^ M and bimolecular rate constants on the order of 10^8^ to 10^10^ M^–1^ s^–1^ (see for example refs (19, 59−61, 66)), rate constants of 10^–10^ to 10^–6^ s^–1^ (t_0.5_ ∼ 10 sunlight days to 300 sunlight years) may be expected.

#### Current Experimental Evidence on (e)DNA Photodegradation

Apart from theoretically evaluating the relevance of the DOM-mediated photodegradation of eDNA, we screened the literature for experimental evidence. While different studies have reported on the overall effect of UV radiation on the persistence of eDNA with contrasting effects (i.e., some studies reporting small to modest UV-induced increases in eDNA loss rates^67−70^ and some reporting no UV-induced effect on loss rates^71−73^), only few studies have analyzed the role of DOM in the photodegradation of eDNA. Three studies by Zhang et al. (2019),^18^ Peng et al. (2023),^59^ and Zhang et al. (2022)^74^ performed direct and ^3^CDOM*-mediated photodegradation experiments of antibiotic resistance genes (ARGs, i.e., specific genomic sequences protecting microbes from antibiotics^75^) contained in plasmid DNA, while three studies by Zhang et al. (2020),^19^ Peng et al. (2024),^60^ and Li et al.^61^ specifically did so for free eDNA (i.e., plasmid DNA, Calf Thymus DNA), free deoxynucleosides (i.e., isolated deoxynucleosides), and free nucleotides (i.e., isolated DNA bases), respectively. The relevant experimental conditions as well as the main results of these studies are briefly summarized in Table 2 (see Tables S3 and S4 for detailed experimental conditions and analytical methods used in these studies to determine the respective damage).

**Table 2:** Summary of the Results of Zhang et al. (2019),^18^ Zhang et al. (2020),^19^ Peng et al. (2023),^59^ Zhang et al. (2022),^74^ Peng et al. (2024),^60^ and Li et al.^61^ on DNA Photodegradation^a^

	Zhang (2019)^18^	Zhang (2020)^19^	Peng (2023)^59^	Zhang (2022)^74^	Peng (2024)^60^	Li (2020)^61^
Direct PD	yes	yes	negl.	yes	very slow	yes
DOM enhanced overall PD	yes	yes	yes (EfOM)	yes	yes	yes
DOM enhanced DD	dG	dG > dA, dT, dC	dG > dA, dT, dC	dG > dA, dT, dC	dG > dA, dT, dC	-
DOM enhanced BD	-	-	-	G, T, C	-	G > A, T, C
DOM enhanced SB	yes	yes	yes	-	-	-
HO· participation	SB	SB, DD	negl.	yes	DD	BD
^1^O_2_ participation	DD (dG)	DD (dG)	negl.	yes	DD (dG)	BD (G)
^3^CDOM* participation	no	-	yes (EfOM)	yes	yes	negl.
DNA substrate	ARG	eDNA	ARG	ARG (PD, DD), DNA bases (BD)	free deoxynucleo-sides	free nucleotides
DOM source	SRNOM	SRNOM	SRNOM, SRFA, SRHA, EfOM	SRFA	SRNOM, EfOM	SRNOM
Wavelength range (nm)	290–400	290–400	AS (>315)	AS (>290)	AS (>315)	290–400

a PD = photodegradation, DD = deoxynucleoside damage (i.e., dG = deoxyguanosine, dA = deoxyadenosine, dT = deoxythymidine, dC = deoxycytidine), BD = base damage (i.e., G = guanine, A = adenine, T = thymine, C = cytosine), SB = strand breaks, ARG = antibiotic resistance gene, EfOM = effluent organic matter, SRNOM = Suwannee River Natural Organic Matter, SRHA = Suwannee River Humic Acid, SRFA = Suwannee River Fulvic Acid, AS = artificial sunlight, negl. f negligible. The experimental conditions and analytical methods used by the studies to determine PD, DD, BD, and SB are specified in Table S3 and S4 in the SI.

While ARGs, plasmid DNA, free deoxynucleosides, or nucleotides differ from eDNA, we view them as appropriate proxies, given their similar structural basis. However, quantitative differences are expected for several reasons. These reasons include the fact that longer DNA strands have a higher probability of damage.^76,77^ Also, different base composition (e.g., AT:GC ratio, sequence) affects potential targets.^76,77^ Finally, conformational differences may lead to different steric accessibility of the nucleotides.^78^ Despite these differences, we view the selected studies as giving a good reflection of the qualitative degradation pathways that can be expected for eDNA and with that of the above hypothesized relevance of DOM-mediated photodegradation in overall eDNA degradation dynamics. Apart from ARGs, plasmid DNA, free deoxynucleosides, or nucleotides, we also considered microbial source tracking markers as proxies. While there is good evidence that sunlight can be an important factor in the fate of fecal microbiota,^79^ we are unaware of studies that examine the photochemical mechanisms at the level of the six studies we have chosen to highlight in Table 2. To accommodate for the fact that most of the six studies did not use eDNA in their degradation experiments, we use the term “DNA” in the subsequent discussion.

Besides the DNA substrate, the source of DOM as well as the wavelength range used for irradiating the samples differed in the experimental setup of the six studies (Table 2). Zhang et al. (2019),^18^ Zhang et al. (2020),^19^ Zhang et al. (2022),^74^ and Li et al.^61^ exclusively used one type of terrestrial DOM in their experiments (SRNOM or SRFA for Zhang et al. (2022)^74^) and employed a wavelength range of 290 to 400 nm or artificial sunlight with a 290 nm cutoff in case of Zhang et al. (2022),^74^ while experiments by Peng et al. (2023)^59^ or Peng et al. (2024)^60^ were performed on terrestrial DOM (SRFA, SRNOM, SRHA or only SRNOM) as well as effluent-derived and thus rather autochthonous DOM (EfOM) using artificial sunlight with a 315 nm cutoff.

As evident from Table 2, four of the six studies observed direct photodegradation of the DNA substrate. DNA and deoxynucleosides mainly absorb UVB light below 300 nm and Peng et al. (2023)^59^ as well as Peng et al. (2024)^60^ used artificial sunlight with a 315 nm cutoff as irradiation source, which explains why direct photodegradation was negligible to absent in their experiments.^59,60^

Compared to direct photodegradation, all studies observed enhanced overall photodegradation in the presence of DOM, even though Peng et al. (2023)^59^ only observed this enhancement for EfOM but not for any of the terrestrial DOM sources SRNOM, SRFA, and SRHA. Since direct photodegradation was absent in their experiments, they hypothesized that compared to other studies, photodegradation rates were not enhanced upon addition of terrestrial DOM because synergistic effects of direct photodegradation and degradation caused by photochemically produced reactive intermediates upon addition of terrestrial DOM may have been absent.^59^ For DNA strands (i.e., ARGs in plasmid DNA, eDNA), the observed reaction rates in the presence of DOM were, depending on the study, enhanced by a factor of 1.4 to 2.0, while they were so by a factor of 12.5, 7.5, 4.1, and 4.3 for the nucleotides guanine, adenine, thymine, and cytosine.^18,19,61,74^ Concerning deoxynucleosides, the enhancement factor was, depending on the DOM source and the deoxynucleoside, between 4 and 72 (EfOM) or 5 and 25 (SRNOM).^60^ We hypothesize that the greater acceleration of the degradation rates observed for the deoxynucleosides and nucleotides upon addition of DOM may be related to differences in their steric accessibility to photochemically produced reactive intermediates from CDOM, compared to bases incorporated into DNA strands.

Concerning specifically observed DNA photodamage, all studies reported deoxynucleoside damage as well as strand breaks, except for Zhang et al. (2022)^74^ who did not report on strand breaks, as well as Peng et al. (2024)^60^ and Li et al.^61^ who analyzed the damage to free deoxynucleosides or nucleotides, respectively. Concerning deoxynucleoside damage, all studies measured selective and significant enhancement of deoxyguanosine photodegradation in the presence of DOM. Likewise, experiments by Li et al.^61^ showed that photodegradation of guanine was most enhanced in the presence of DOM. Finally, all studies that included strand breaks in their analysis^18,19,59^ observed significant enhancement of strand breaks in the presence of DOM.

Regarding participation of ^3^CDOM*, HO·, and ^1^O_2_ in specific photodegradation reactions, the studies agree on ^1^O_2_ being primarily involved in the oxidation of deoxyguanosine or guanine, while HO· was, depending on the study, found to induce strand breaks or deoxynucleoside as well as nucleotide damage. Concerning ^3^CDOM*, however, the results of the studies differ significantly even for experiments using the same type of DOM. All experiments using SRNOM observed no ^3^SRNOM* participation in DNA degradation, except for Peng et al. (2024)^60^ who attributed 15% of the overall deoxyguanosine degradation to ^3^SRNOM*. Furthermore, experiments by Peng et al. (2023),^59^ Peng et al. (2024)^60^ or Zhang et al. (2022)^74^ using EfOM or SRFA found ^3^CDOM* to participate in the degradation of their DNA substrate. In case of Peng et al. (2023)^59^ or Peng et al. (2024),^60^^3^EfOM* even dominated overall DNA or deoxyguanosine degradation, while for Zhang et al. (2022)^74^^3^CDOM*-mediated photodegradation contributed most to DNA degradation after direct photodegradation (39.8% vs 60.0%).

Peng et al. (2023)^59^ performed consecutive experiments, shining light on the differences in the photoreactivity observed for terrestrial- and effluent-derived DOM. To do so, they characterized the energy level of the excited triplet states of the DOM sources and further analyzed the reactivity of model sensitizers of various excited triplet state energies with ARGs. The latter showed that ARG degradation was fastest for model sensitizers with high triplet energies (>280 kJ/mol) and high reduction potentials (>1.7 vs NHE), present but slower for those with either high triplet energies (>184 kJ/mol) or high reduction potentials (>1.47 vs NHE) and absent for model sensitizers with both low triplet energies and reduction potentials.^59^ Together with the fact that effluent derived DOM was found to be much more efficient at forming high-energy triplets (∼78% of all excited triplet states) than terrestrially derived DOM (∼30–59% of all excited triplet states),^59^ the differences in the photoreactivity of terrestrial DOM vs EfOM observed by Peng et al. (2023)^59^ indicate that the energy level as well as the reduction potential of the respectively formed excited triplet state may determine the photoreactivity of ^3^CDOM* with ARGs and thus eDNA.

To recap, the main takeaways of our earlier theoretical evaluation of indirect photodegradation pathways of eDNA were:

(1) The reduction potential of ^3^CDOM* is thermodynamically high enough to induce oxidation of (e)DNA,
(2) HO· is capable of inducing strand breaks as well as oxidative modifications of (e)DNA nucleotides,
(3) ^1^O_2_ can oxidize guanine contained in (e)DNA.

The predictions of ^3^CDOM*, HO·, and ^1^O_2_ participation in eDNA photodegradation align well with the few experimental studies that have been conducted. This experimental insight further emphasizes the diverse photochemical characteristics of ^3^CDOM* that allow it to promote reactions with a wide range of substrates including eDNA.

## Implications

The presented findings highlight the potential role of photochemistry in determining the fate of eDNA. The literature review on DOM degradation emphasizes that photochemistry may be the main driver of eDNA degradation under certain environmental conditions (e.g., CDOM-rich waters with no light-limitation and low temperature water bodies with low microbial activity) and may further influence microbial degradation rates of eDNA. Furthermore, ^3^CDOM* and ROS likely participate in eDNA degradation processes in sunlit surface waters, which may significantly contribute to the overall degradation of eDNA observed in aquatic ecosystems. These findings necessitate the clarification of the role of photochemistry and specifically that of ^3^CDOM* in eDNA degradation processes by future studies, which will eventually allow for more reliable spatiotemporal inferences of species prevalence from eDNA analyses.

To clarify the role of photochemistry in eDNA degradation dynamics, future studies should consider that

(1) eDNA is susceptible to both direct and indirect photochemical reactions and that such reactions will affect eDNA persistence in sunlit waters.
(2) there is a need for more experiments assessing the photochemical impact on eDNA as well as comparing the contributions of photochemical and microbial processes to eDNA degradation, similar to those performed on DOM degradation. This necessity is further highlighted by the fact that the contributions by Zhang (2019)^18^ and Zhang (2020)^19^ or Peng (2023)^59^ and Peng (2024)^60^ listed in Table 2 were produced by the same laboratory group.
(3) it remains unknown whether photochemical reactions primarily lead to the breakdown (i.e., fragmentation, mineralization) or alteration (e.g., oxidative damage to eDNA nucleotides) of the eDNA molecule. It should further be assessed whether and if so to what extent photoalterations of eDNA affect the detection of eDNA through (q)PCR.
(4) the impact of photochemistry will differ in the different layers of a stratified water body. This in turn may impact species-specific detection limits in these layers and should inform sampling strategies. More experiments are needed to verify this expected effect and to quantify its magnitude.
(5) there is a need to resolve the impact of differently sourced DOM and thereof produced reactive chemical species on eDNA degradation.
(6) the degradation dynamics of eDNA should be examined for different types of aquatic ecosystems such as lakes, rivers, brackish waters, or the ocean. In marine systems, an important factor to consider is that reactive halogen species are also expected to play a role in the photodegradation of eDNA.^80^
(7) photochemically induced eDNA alterations, such as the oxidation and dimerization of DNA bases as well as single-strand breaks, may lead to negative detection events in eDNA analyses, as the binding of primers or the amplification efficiency during qPCR could be affected in these damaged regions. Peng et al. (2024)^60^ showed that in the case of DNA-bound nucleobases, guanine is most susceptible to photodegradation in the presence of effluent DOM compared to the other nucleobases.^60^ Similarly, Shin and Lee^76^ observed that the degradation rates of an ampicillin resistance gene via ^1^O_2_ increased with an increasing number of guanine bases contained in the amplicon region.^76^ Due to this high oxidation susceptibility of DNA-bound guanine, primers targeting eDNA regions rich in guanine and cytosine may lead to lower detection probabilities of eDNA compared to primers targeting adenine-thymine-rich sequences. The high oxidative susceptibility of guanine may also lower the detection probabilities of guanine- and cytosine-rich biological species compared to those rich in adenine and thymine. Such dynamics may further be relevant to explore for primers or species rich in adjacent thymine bases, since results by Dunn and Silverman suggest that the decay rates of ARGs quantified by qPCR may depend on the number of potential CPD formation sites.^77^ Integrating these aspects into the design of primers and regions of genomes targeted for eDNA analyses may improve general as well as biological species-specific detection probabilities and maybe even more so in combination with efficient eDNA repair treatments, restoring those photochemically induced eDNA alterations. What we observe as a biological species-specific effect on the detection of eDNA may in fact be resolved by this photodegradation mechanism because base composition differs across the tree of life.^81^

## Supporting Information Available

Geographical location of the DOM studies discussed in Table 1 (Figure S1), exemplary UV–vis absorption spectra of DOM isolates and eDNA (Figure S2, S3), detailed experimental conditions and analytical methods that were used in the DOM and eDNA studies discussed in Tables 1 and 2 (Tables S1–S4) (PDF)
