# Supplementary material for: Potential Role of Photochemistry in Environmental DNA Degradation
Source: Environ Sci Technol Lett. 2024 Nov 26;11(12):1284–95. doi: 10.1021/acs.estlett.4c00704 (PMC11636254; doi:10.1021/acs.estlett.4c00704)
Supplement: Supplementary file 1 — ez4c00704_si_001.pdf [file ez4c00704_si_001.pdf]

Supporting Information

The Potential Role of Photochemistry in Environmental DNA Degradation

Eliane Ballmer, Kristopher McNeill*, Kristy Deiner*

Institute of Biogeochemistry and Pollutant Dynamics, ETH Zurich, 8092 Zurich, Switzerland

Contains:

3 Figures

4 Tables

Study Sites

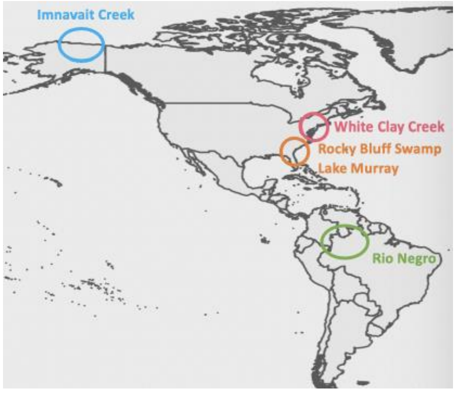

Figure S1: Geographical location of the four studies discussed in Table 1 and S1.[1–4]

Experimental Conditions Used in DOM Studies

Table S1: Experimental conditions of the four studies compared in Table 1 (a = autochthonous, t = terrestrial, (t) = the respective study did not specify the type of DOM, wherefore it was inferred from the information provided by the study, T = tropic, ST = subtropic, A = Arctic, TE = temperate, light = characterization of experimental irradiation source, NS = natural sunlight, SS = solar simulator, T/t_{irr} = irradiation temperature and time, T/t_{inc} = incubation temperature and time).[1–4]

DOM source	DOM type (-)	Habitat (-)	Climate (-)	[DOC] (mM)	Light (-)	t_{irr} (h)	t_{inc} (d)	T_{irr} (°C)	T_{inc} (°C)
Rio Negro[1]	(t)	river	T	0.83	NS	4-27	0.5-1	28	-
Rocky Bluff Swamp[2]	t	swamp	ST	1.07	SS	201	111	20	25
Lake Murray[2]	**t**,a	lake	ST	0.32	SS	117	297	20	20
Phytoplankton[2]	a	tank	ST	0.07	SS	12	117	20	20
Imnavait Creek[3]	t	creek	A	0.8-1.2	NS	12	5-7	10-16	6-7
White Clay Creek[4]	**t**,a	creek	TE	0.13	NS	3-17	<0.03	27-32	20

Analytical Methods Used in DOM Studies

Table S2: Summary of the analytical methods used by Amon and Benner[1], Obernosterer and Benner[2], Cory et al.[3] and Bowen et al.[4] to assess the relative importance of photochemical (PD) vs. microbial (MD) degradation processes (i.e., mineralization to CO_2) in overall DOM degradation dynamics (DOC = dissolved organic carbon, $O_{2,diss}$ = dissolved oxygen, DIC = dissolved inorganic carbon).

DOM source	PD	MD
Rio Negro[1]	DOC[i)], $O_{2,diss}$[ii)]	DOC[i)], $O_{2,diss}$[ii)]
Rocky Bluff Swamp[2]	DOC[i)]	DOC[i)]
Lake Murray[2]	DOC[i)]	DOC[i)]
Phytoplankton[2]	DOC[i)]	DOC[i)]
Imnavait Creek[3]	DIC[iii)]	DIC[iii)]
White Clay Creek[4]	DOC[i)]	DOC[i)]

[i)]DOC was measured using a total organic carbon (TOC) analyzer.
[ii)]$O_{2,diss}$ was determined using the Winkler method.
[iii)]DIC was measured using a DIC analyzer.

DNA Absorption Spectrum

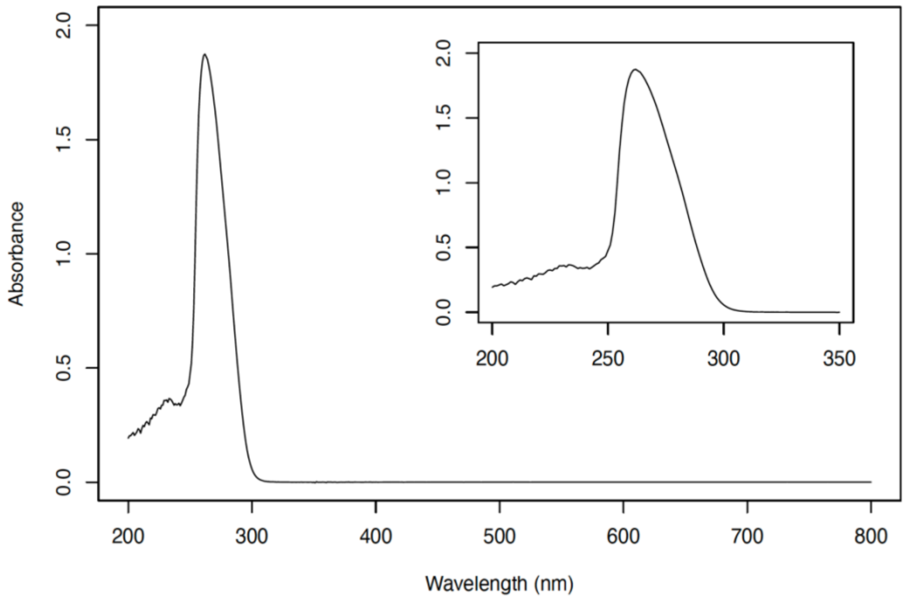

Figure S2: UV-Vis absorption spectrum of sheared salmon sperm DNA (Invitrogen, 100 mg DNA/L, Longmire buffer) collected with a Varian Cary 100 Bio Spectrophotometer.

DOM Absorption Spectrum

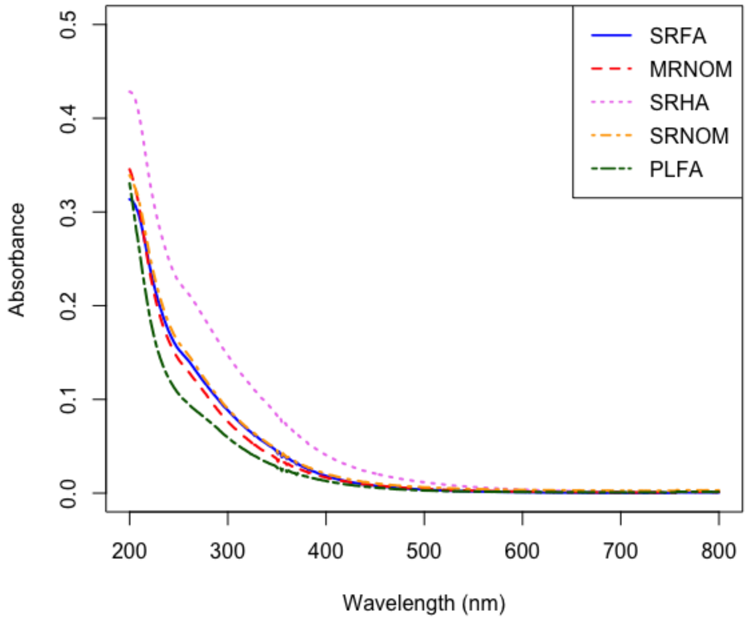

Figure S3: UV-Vis absorption spectra of DOM isolates obtained from the International Humic Substances Society, prepared in phosphate buffer (pH 7) and measured using a Varian Cary 100 Bio Spectrophotometer: Suwannee River Fulvic Acid (SRFA, 3.1 mgC/L, Standard II, 2S101F), Upper Mississippi River Natural Organic Matter (MRNOM, 4.3 mgC/L, 1R110N), Suwannee River Humic Acid (SRHA, 3.7 mgC/L, 2S101H), Suwannee River Natural Organic Matter (SRNOM, 3.9 mgC/L, 2R101N), Pony Lake Fulvic Acid (PLFA, 4.2 mgC/L, 1R109F).

Experimental Conditions Used in eDNA Studies

Table S3: Summary of the experimental conditions of experiments performed by Zhang et al. (2019)[5], Zhang et al. (2020)[6], Peng et al. (2023)[7], Zhang et al. (2022)[8], Peng et al. (2024)[9] and Li et al.[10] on (e)DNA photodegradation (PPRI = photochemically produced reactive intermediate, OPD = overall photodegradation, DD = deoxynucleoside damage, BD = base damage, SB = strand breaks, ARG = antibiotic resistance gene, EfOM = effluent organic matter, SRNOM = Suwannee River natural organic matter, SRHA = Suwannee River humic acid, SRFA = Suwannee River fulvic acid, AS = artificial sunlight, PB = phosphate buffer, SA = short-amplicon quantitative polymerase chain reaction, LA = long-amplicon quantitative polymerase chain reaction).

	Zhang (2019)[5]	Zhang (2020)[6]	Peng (2023)[7]	Zhang (2022)[8]	Peng (2024)[9]	Li (2020)[10]
Analyzed damage	OPD, DD, SB	OPD, DD, SB	OPD, DD, SB	OPD, DD, BD	DD	BD
Analyzed PPRI	^{3}SRNOM*, HO·, $^1O_2$	HO·, $^1O_2$	^{3}EfOM*, HO·, $^1O_2$, $O_2^{\cdot -}$	^{3}SRFA*, HO·, $^1O_2$	^{3}EfOM*, ^{3}SRNOM*, HO·, $^1O_2$, $O_2^{\cdot -}$	^{3}SRNOM*, HO·, $^1O_2$, $O_2^{\cdot -}$
DNA substrate	ARG	eDNA	ARG	ARG	free deoxynucleo-sides	free nucleotides
DOM source	SRNOM	SRNOM	SRNOM, SRFA, SRHA, EfOM	SRFA	EfOM, SRNOM	SRNOM
Light source	Mercury lamp	Mercury lamp	Xenon lamp	Xenon lamp	Xenon lamp	Mercury lamp
Wavelength range (nm)	290-400	290-400	AS (>315)	AS (>290)	AS (>315)	290-400
[DOM]	0-50mg/L	0-50mg/L	0-5mgC/L	4.8 mgC/L	5 mgC/L	0-50mg/L
Medium	PB (20mM)	PB (50mM)	water	PB (NA)	PB (5mM)	PB (10mM)
pH	7.3	7	6.5	7	7	7
T (°C)	25	25	25	25	25	25
Time points	5[a)]	5[a)]	6[a)]	6	6[a)]	5[a)]
Duration (h)	SA: 0-12[a)] LA: 0-4[a)]	0-70[a)]	0-4[a)]	0-4	0-48[a)]	0-ca.22[a)]

a) The number of time points and the duration of the last time point were estimated from the figures in the respective publications, including the 0-time point.

Analytical Methods Used in eDNA Studies

Table S4: Summary of analytical methods used by Zhang et al. (2019)[5], Zhang et al. (2020)[6], Peng et al. (2023)[7], Zhang et al. (2022)[8], Peng et al. (2024)[9], and Li et al.[10] to asses overall photodegradation (OPD), deoxynucleoside damage (DD), base damage (BD) and strand breaks (SB) (ARG = antibiotic resistance gene, qPCR = quantitative polymerase chain reaction, HPLC-UV = high-performance liquid chromatography coupled to an ultraviolet (UV) detector, HPLC-DAD = HPLC coupled to a diode array detector, HPLC-MS/MS = HPLC-tandem mass spectrometry, HPLC-/LC-MS = HPLC/liquid chromatography (LC)-mass spectrometry, GE = gel electrophoresis). All ARGs were studied as part of plasmid DNA.

	Zhang (2019)[5]	Zhang (2020)[6]	Peng (2023)[7]	Zhang (2022)[8]	Peng (2024)[9]	Li (2020)[10]
Analyzed damage	OPD, DD, SB	OPD, DD, SB	OPD, DD, SB	OPD, DD, BD	DD	BD
DNA substrate	ARG: *tet A* *bla*$_{TEM-1}$	plasmid DNA (OPD, SB) Calf thymus DNA (DD)	ARG: *tet A* *bla*$_{TEM-1}$	ARG (OPD, DD): *tet A* DNA bases (BD)	deoxy-nucleosides	nucleo-tides
OPD	qPCR[1]	qPCR[2]	qPCR[3]	qPCR[4]	-	-
DD	HPLC-UV (-MS/MS)[5]	HPLC-UV, (LC-MS)[5]	HPLC-DAD	HPLC-UV (-MS)[5]	HPLC-DAD (-MS)[5]	-
SB	GE	GE	GE	-	-	-
BD	-	-	-	HPLC-UV	-	HPLC-UV (-MS)[5]

[1]The ARGs were quantified using short- (~200 base pairs) and long- (whole gene) amplicon qPCR.

Forward and reverse primer sequence *tet A*:

Short-amplicon: GACTATCGTCGCCGCACTTA; ATAATGGCCTGCTTCTCGCC

Long-amplicon: CGTGTATGAAATCTAACAATGCGCT; CCATTCAGGTCGAGGTGGC

Forward and reverse primer sequence *bla*$_{TEM-1}$:

Short-amplicon: AATAAACCAGCCAGCCGGAA; TTGATCGTTGGGAACCGGAG

Long-amplicon: TTACCAATGCTTAATCAGTGAGGC; ATGAGTATTCAACATTTCCGTGTCG

[2]The primers used for qPCR covered 216 base pairs of the plasmid DNA. The forward and reverse primer sequence matches that of the primer used for short-amplicon qPCR of *tet A* referenced in footnote 1.

[3]The ARGs were quantified using short-amplicon qPCR (*tet A*: 216 base pairs, *bla*$_{TEM-1}$: 209 base pairs). The forward and reverse primer sequence matches that of the primer used for short-amplicon qPCR of *tet A* and *bla*$_{TEM-1}$ referenced in footnote 1.

[4]The forward and reverse sequence of the short-amplicon qPCR primer matches that of the short-amplicon qPCR of *tet A* referenced in footnote 1.

[5]HPLC-UV/-DAD was used to quantify DD or BD, while (HP)LC-MS(/MS) was used to identify respective photoproducts.

References

(1) Amon, R. M. W.; Benner, R. Photochemical and Microbial Consumption of Dissolved Organic Carbon and Dissolved Oxygen in the Amazon River System. *Geochim. Cosmochim. Acta* **1996**, *60* (10), 1783–1792. https://doi.org/10.1016/0016-7037(96)00055-5.

(2) Obernosterer, I.; Benner, R. Competition between Biological and Photochemical Processes in the Mineralization of Dissolved Organic Carbon. *Limnol. Oceanogr.* **2004**, *49* (1), 117–124. https://doi.org/10.4319/lo.2004.49.1.0117.

(3) Cory, R. M.; Harrold, K. H.; Neilson, B. T.; Kling, G. W. Controls on Dissolved Organic Matter (DOM) Degradation in a Headwater Stream: The Influence of Photochemical and Hydrological Conditions in Determining Light-Limitation or Substrate-Limitation of Photo-Degradation. *Biogeosciences* **2015**, *12* (22), 6669–6685. https://doi.org/10.5194/bg-12-6669-2015.

(4) Bowen, J. C.; Kaplan, L. A.; Cory, R. M. Photodegradation Disproportionately Impacts Biodegradation of Semi-Labile DOM in Streams. *Limnol. Oceanogr.* **2020**, *65* (1), 13–26. https://doi.org/10.1002/lno.11244.

(5) Zhang, X.; Li, J.; Fan, W. Y.; Yao, M. C.; Yuan, L.; Sheng, G. P. Enhanced Photodegradation of Extracellular Antibiotic Resistance Genes by Dissolved Organic Matter Photosensitization. *Environ. Sci. Technol.* **2019**, *53* (18), 10732–10740. https://doi.org/10.1021/acs.est.9b03096.

(6) Zhang, X.; Li, J.; Yao, M.-C.; Fan, W.-Y.; Yang, C.-W.; Yuan, L.; Sheng, G.-P. Unrecognized Contributions of Dissolved Organic Matter Inducing Photodamages to the Decay of Extracellular DNA in Waters. *Environ. Sci. Technol.* **2020**, *54* (3), 1614–1622. https://doi.org/10.1021/ACS.EST.9B06029.

(7) Peng, J.; Pan, Y.; Zhou, Y.; Kong, Q.; Lei, Y.; Lei, X.; Cheng, S.; Zhang, X.; Yang, X. Triplet Photochemistry of Effluent Organic Matter in Degradation of Extracellular Antibiotic Resistance Genes. *Environ. Sci. Technol.* **2023**, *57*, 7230–7239. https://doi.org/10.1021/acs.est.2c08036.

(8) Zhang, T.; Cheng, F.; Yang, H.; Zhu, B.; Li, C.; Zhang, Y. nan; Qu, J.; Peijnenburg, W. J. G. M. Photochemical Degradation Pathways of Cell-Free Antibiotic Resistance Genes in Water under Simulated Sunlight Irradiation: Experimental and Quantum Chemical Studies. *Chemosphere* **2022**, *302*, 134879. https://doi.org/10.1016/j.chemosphere.2022.134879.

(9) Peng, J.; Pan, Y.; Zhou, Y.; Lei, X.; Guo, Y.; Lei, Y.; Kong, Q.; Cheng, S.; Yang, X. Mechanistic Aspects of Photodegradation of Deoxynucleosides Induced by Triplet State of Effluent Organic Matter. *Environ. Sci. Technol.* **2024**, *58* (10), 4751–4760. https://doi.org/10.1021/acs.est.3c08782.

(10) Li, J.; Zhang, X.; Fan, W. Y.; Yao, M. C.; Sheng, G. P. Dissolved Organic Matter Dominating the Photodegradation of Free DNA Bases in Aquatic Environments. *Water Res.* **2020**, *179*. https://doi.org/10.1016/j.watres.2020.115885.
